# Implementing Standard Diagnosis and Treatment for Locally Advanced Breast Cancer Through Global Research in Latin America: Results From a Multicountry Pragmatic Trial

**DOI:** 10.1200/GO.23.00216

**Published:** 2024-05-09

**Authors:** Javier Retamales, Adrián Daneri-Navarro, Nora Artagaveytia, Daniela Alves da Quinta, Eliana Abdelhay, Osvaldo L. Podhajcer, Carlos Velázquez, Diego Giunta, Susanne Crocamo, Adriana Garibay-Escobar, Alicia Del Toro-Arreola, Robinson Rodriguez, Marta Aghazarian, Elsa Alcoba, Isabel Alonso, Renata Binato, Alicia I. Bravo, Juan Canton-Romero, Dirce M. Carraro, Mónica Castro, Juan Castro-Cervantes, Sandra Cataldi, Natalia Camejo, Laura Cortes-Sanabria, Maria Flores-Marquez, Guillermo Laviña, Eduardo Musetti, Benedicta Caserta, Mauricio Cerda, Alicia Colombo, Raul Delgadillo-Cristerna, Marisa Dreyer Breitenbach, Elmer Fernandez, Jorge Fernandez, Ramon Franco-Topete, Carolina Gabay, Fancy Gaete, Jorge Gamboa, Ricardo García-Gaeta, Mariana Gomez del Toro, Leivy P. Gonzalez-Ramirez, Marisol Guerrero, Manuel Herrera-Miramontes, Alejandra Lopez-Vasquez, Silvina Maldonado, Andrés Morán-Mendoza, Gilberto Morgan-Villela, Maria Aparecida Nagai, Nancy Navarro-Ruiz, Antonio Oceguera-Villanueva, Miguel Angel Ortiz, Jael Quintero, Antonio Quintero-Ramos, Gladys Ramirez-Rosales, Maritza Ramos-Ramirez, Marcia Maria Chiquitelli Marques, Ernesto Rivera Claisse, Diego Rodriguez-Gonzalez, Ana Romero-Gomez, Cristina Rosales, Efraín Salas-Gonzalez, Verónica Sanchotena, Laura Segovia, Aída A. Silva-García, Olivia Valenzuela-Antelo, Laura Venegas-Godinez, Livia Zagame, Jorge Gomez, Andrea S. Llera, Bettina Müller

**Affiliations:** ^1^ Grupo Oncologico Cooperativo Chileno de Investigacion, Santiago, Chile; ^2^ Universidad de Guadalajara, Guadalajara, Mexico; ^3^ Hospital Universitario de Clínicas “Manuel Quintela,” Montevideo, Uruguay; ^4^ Fundación Instituto Leloir-CONICET, Buenos Aires, Argentina; ^5^ Universidad Argentina de la Empresa (UADE), Instituto de Tecnología (INTEC), Buenos Aires, Argentina; ^6^ Instituto Nacional de Câncer Rio de Janeiro, Rio de Janeiro, Brazil; ^7^ Universidad de Sonora, Sonora, Brazil; ^8^ Instituto Universitario Hospital Italiano de Buenos Aires-CONICET, Buenos Aires, Argentina; ^9^ Instituto Nacional de Cancer, Montevideo, Uruguay; ^10^ Hospital Municipal de Oncología María Curie, Buenos Aires, Argentina; ^11^ Centro Hospitalario Pereira Rossell, Montevideo, Uruguay; ^12^ Hospital Regional de Agudos Eva Perón, Buenos Aires, Argentina; ^13^ Hospital de Gineco-Obstricia CMNO-IMSS, Guadalajara, Mexico; ^14^ AC Camargo Cancer Center, São Paulo, Brazil; ^15^ Instituto de Oncología Angel Roffo, Buenos Aires, Argentina; ^16^ Hospital de Especialidades CMNO-IMSS, Guadalajara, Mexico; ^17^ Universidad de Chile, Santiago, Chile; ^18^ Universidade Estadual do Rio de Janeiro, Rio de Janeiro, Brazil; ^19^ Fundación para el Progreso de la Medicina, Cordoba, Argentina; ^20^ Instituto de Salud Publica, Santiago, Chile; ^21^ OPD Hospital Civil de Guadalajara, Guadalajara, Mexico; ^22^ Hospital Luis Tisne, Santiago, Chile; ^23^ Hospital Clínico San Borja Arriaran, Santiago, Chile; ^24^ Hospital San Jose, Santiago, Chile; ^25^ Instituto de Câncer de São Paulo, São Paulo, Brazil; ^26^ Instituto Jalisciense de Cancerologia, Guadalajara, Mexico; ^27^ Hospital General Regional No. 1, IMSS Obregon, Sonora, Mexico; ^28^ Hospital de Câncer de Barretos, Barretos, Brazil; ^29^ Centro Estatal de Oncología, Hermosillo, Mexico; ^30^ Hospital Barros Luco Trudeau, Santiago, Chile; ^31^ Health Sciences Center, Texas A&M University, College Station, TX; ^32^ Instituto Nacional del Cáncer, Santiago, Chile

## Abstract

**PURPOSE:**

Breast cancer mortality rates in Latin America (LA) are higher than those in the United States, possibly because of advanced disease presentation, health care disparities, or unfavorable molecular subtypes. The Latin American Cancer Research Network was established to address these challenges and to promote collaborative clinical research. The Molecular Profiling of Breast Cancer Study (MPBCS) aimed to evaluate the clinical characteristics and treatment outcomes of LA participants with locally advanced breast cancer (LABC).

**PATIENTS AND METHODS:**

The MPBCS enrolled 1,449 participants from Argentina, Brazil, Chile, Mexico, and Uruguay. Through harmonized procedures and quality assurance measures, this study evaluated clinicopathologic characteristics, neoadjuvant chemotherapy response, and survival outcomes according to residual cancer burden (RCB) and the type of surgery.

**RESULTS:**

Overall, 711 and 480 participants in the primary surgery and neoadjuvant arms, respectively, completed the 5-year follow-up period. Overall survival was independently associated with RCB (worse survival for RCBIII-adjusted hazard ratio, 8.19, *P* < .001, and RCBII [adjusted hazard ratio, 3.69, *P* < .008] compared with RCB0 [pathologic complete response or pCR]) and type of surgery (worse survival in mastectomy than in breast-conserving surgery [BCS], adjusted hazard ratio, 2.97, *P* = .001). The hormone receptor–negative-human epidermal growth factor receptor 2–positive group had the highest proportion of pCR (48.9%). The analysis of the ASCO Quality Oncology Practice Initiative breast module revealed high compliance with pathologic standards but lower adherence to treatment administration standards. Notably, compliance with trastuzumab administration varied widely among countries (33.3%-88.7%).

**CONCLUSION:**

In LABC, we demonstrated the survival benefit of BCS and the prognostic effect of the response to available neoadjuvant treatments despite an important variability in access to key treatments. The MPBCS represents a significant step forward in understanding the real-world implementation of oncologic procedures in LA.

## Teaser

Involving over 30 clinical and research institutions, the LACRN not only enrolled 1,449 participants from Argentina, Brazil, Chile, Mexico, and Uruguay for the study but also implemented standardized diagnostic and treatment procedures.

## INTRODUCTION

Breast cancer incidence in Latin America (LA) is lower than that in the United States, but the mortality rate is higher.^1^ Potential reasons include late-stage clinical presentation, health care access disparities, and higher proportion of molecular subtypes with poor prognosis. Locally advanced breast cancer (LABC) is typically categorized as either stage II or III.^2^ LA countries have a higher proportion of LABC than developed nations, ranging from 15% to 30%.^3^ By contrast, data from the National Cancer Database and CONCORD study suggest that 8.5% of American and 4% of European participants with breast cancer present with LABC,^4^ raising concerns regarding health care accessibility for the LA population.

**CONTEXT**

**Key Objective**
How do standardized diagnosis and treatment for locally advanced breast cancer (LABC) affect patient outcomes in Latin America, given the region's unique challenges and disparities?
**Knowledge Generated**
The Molecular Profiling of Breast Cancer Study enrolled more than 1,400 participants from five Latin American countries. The data showed that survival was significantly influenced by residual cancer burden and the type of surgery performed. Hormone receptor–negative-human epidermal growth factor receptor 2–positive patients exhibited the highest proportion of complete response. Treatment administration standards showed variability, with notable discrepancies in trastuzumab administration across countries.
**Relevance**
Establishing and adhering to standardized diagnostic and treatment protocols can substantially influence LABC outcomes in LA. The findings underscore the significance of consistent access to standard-of-care therapies in enhancing patient survival and emphasize the role of collaborative clinical research in addressing health care disparities.

Treatment of LABC involves combined systemic therapies (chemotherapy, endocrine therapy, and antihuman epidermal growth factor receptor 2 [HER2] therapies) and locoregional modalities (surgery and radiotherapy) through a coordinated multidisciplinary approach. An accurate histologic diagnosis, including immunohistochemistry (IHC) and HER2 amplification tests, is essential.^5^ LA countries differ in socioeconomic factors, public health infrastructure, ethnicity, and culture, resulting in disparities in access to those procedures.^6^

To address the cancer burden and establish a comprehensive clinical research network, the US-National Cancer Institute's Center of Global Health collaborated with LA countries' Ministries of Health or Science to launch the LA-Cancer Research Network (LACRN).^7^ LACRN established standardized activities across 27 clinical centers, six universities, and five research institutions in Argentina, Brazil, Chile, Mexico, and Uruguay.^7^ Its first study, the Molecular Profiling of Breast Cancer Study (MPBCS), was launched to investigate patterns of care and molecular subtypes of LABC in LA. The MPBCS enrolled 1,449 participants collecting molecular, clinical, and epidemiologic data through harmonized procedures. The initial publication revealed that overall survival (OS) was associated with molecular subtypes and that molecular profiling demonstrated similarities to stage-matched The Cancer Genome Atlas (TCGA) participants,^8^ suggesting that higher mortality in LA may not be primarily due to molecular differences.

This report focused on the clinical characteristics and OS outcomes in MPBCS. We analyzed the response to neoadjuvant chemotherapy (NAC) using residual cancer burden (RCB) index^9^ and its association with survival. In addition, we investigated OS outcomes on the basis of the type of surgery, either total mastectomy or breast-conserving surgery (BCS). An exploratory analysis using the ASCO Quality Oncology Practice Initiative (QOPI)^10^ breast module indicators was conducted to assess harmonization results.

## PATIENTS AND METHODS

### Study Design and Governance

MPBCS was designed as a pragmatic clinical trial.^11^ We use term “pragmatic trial” to reflect the characteristic of evaluating interventions under routine practice conditions rather than controlled clinical settings.^11^ This study was sponsored by the National Cancer Institute (NCI; Project grant No. HHSN2612010000871/NO2-PC-2010-00087) and was conducted under governance of a steering committee.

Study protocol was approved by the NCI Ethics Committee and local institutional review boards in each country, registered at ClinicalTrials.gov (identifier: NCT02326857),^12^ and conducted in accordance with Declaration of Helsinki and local regulations. All participants signed study-specific informed consent form before study procedures.

### Study Participants

Detailed eligibility criteria have been described previously.^7,8,13^ Briefly, women with clinical stage II or III (American Joint Committee on Cancer 7) breast cancer were eligible for this study and were invited to participate by their treating physician who was part of the study team. Women who agreed to participate signed informed consent form and were assigned to one of two primary treatments according to breast surgeon criteria: primary surgery for women who were deemed suitable candidates for immediate surgical resection and primary systemic treatment for women eligible for NAC. Participants with bilateral or inflammatory breast cancer or metastatic disease were excluded.

### Study End Points

The end points reported here were as follows: (1) cohort characteristics: age, menopausal status, IHC-based subtype, PAM50 molecular subtype, and type of primary treatment; (2) response to NAC: proportion of participants achieving a pathological complete response (pCR) or other response, evaluated by the RCB classification and stratified by molecular (PAM50) and IHC subtypes, and OS according to the type of response; and (3) OS outcomes on the basis of the type of surgery, considering both primary and post-NAC surgical procedures.

### Clinical Procedures

#### Primary Surgery

Participants underwent routine clinical evaluation, staging (including computed tomography and bone scans), and surgery (total mastectomy or BCS, with lumpectomy and quadrantectomy as the BCS option). Tissue samples were collected during surgery.

#### Neoadjuvant Treatment

Participants, after routine evaluation and staging, received primary systemic therapy (NAC) followed by surgery (total mastectomy or BCS) within 42 days postchemotherapy. Tissue samples were collected during diagnostic biopsies and surgery.

The choice between BCS and mastectomy was based on surgeon’s discretion and, when applicable, on participant’s preference. Total mastectomy referred to complete removal of breast tissue but did not include more extensive procedures, such as Halsted radical mastectomy. Skin sparing or other types of mastectomies were not differentiated in this analysis. Documentation of negative margins was required for participants who underwent breast preservation. Postoperative radiotherapy was mandatory for BCS.

Tissues were processed according to TCGA-based harmonized standardized operative procedures (SOPs).^8^ Tumor location was marked before NAC.

Estrogen receptor, progesterone receptor, HER2 (by IHC and either fluorescence in situ hybridization [FISH]/chromogenic in situ hybridization [CISH] in indeterminate cases, ie, 2+ by IHC), and Ki67 statuses were determined locally following SOPs.^8^ All local pathology departments were accredited by the College of American Pathologists (CAP).

Recommended regimens for NAC included doxorubicin (60 mg/m^2^) or epirubicin (75-100 mg/m^2^) + cyclophosphamide (600 mg/m^2^) once every 3 weeks for four cycles, followed by either paclitaxel (80 mg/m^2^) once per week for 12 cycles or docetaxel (70-90 mg/m^2^) once every 3 weeks for four cycles. Addition of trastuzumab to participants with HER2+ tumors was strongly recommended.

Participants were followed up for 5 years postoperatively to monitor survival and recurrence. Clinical data were captured using electronic case report forms in OpenClinica, with accuracy ensured by local data managers.

#### Pathologic Response Evaluation

Pathologic responses and RCB evaluations were used to assess surgically resected specimens from participants who received NAC. RCB scores were determined using the MD Anderson algorithm.^9,14^

### Microarray Data Acquisition and PAM50 Subtype Assignation

Detailed information regarding molecular subtype determination has been described previously.^8^ Quality control measures, including principal component analysis, were implemented to avoid bias.

### Association Analysis

Chi-square tests were performed for association analysis between categorical variables throughout the study. Pearson’s residuals were used to represent the degree of association between RCB scores and IHC or PAM50 subtypes. Balloon plots were used to represent the residual values (ggpubr R package). All statistical analyses were performed using R, version 4.1.3, and two-tailed statistical significance was set at *P* < .05.

### Survival Analysis

Survival analyses (survival R package) were performed using the Kaplan-Meier estimator, and curves were compared using the log-rank test. OS was defined as the interval from the date of surgery to the date of death from any cause. This approach minimizes potential immortal time bias that could arise if the interval from the date of diagnosis or surgical biopsy is considered, providing a more accurate estimate for both groups. Participants without a date of death were censored on the off-study period. Univariate Cox proportional hazards regression models were used to estimate hazard ratio (with 95% CIs) using death as the outcome. The proportional hazard assumption was verified using scaled Schoenfeld residuals.

For RCB-related survival analysis, a multivariate Cox proportional hazards regression model was adjusted for age, menopausal status, histologic grade, country, neoadjuvant treatment (yes/no), clinical T stage (cT), clinical nodal status (cN), and IHC subtype.

**FIG A1:**
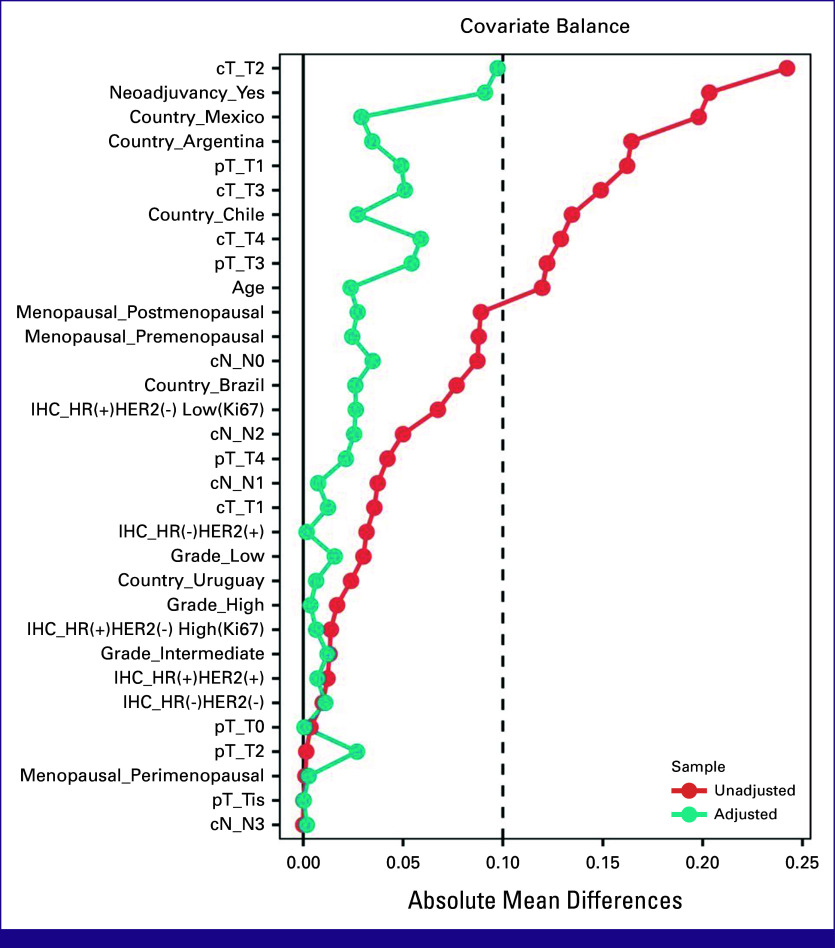
Covariance balance plot for all variables considered in the IPTW model. IPTW, inverse probability of treatment weighting.

For surgical treatment–related survival analysis we used a causal approach to control for confounders, balancing the distribution of variables considered by surgeons to select surgical approach (ie, mastectomy or BCS) and creating a pseudorandomized sample to reduce bias estimating effects. A multivariate Cox proportional hazards regression model weighted by inverse probability of treatment weighting (IPTW) was used (WeightIt R package). Weight of each participant was calculated as inverse of the probability of receiving either mastectomy or BCS as treatment (ie, propensity score), considering as causes of treatment decisions and outcomes the following: age, menopausal status, grade, country, neoadjuvant treatment, cT, cN, pathologic T stage (pT: T0-T4 and Tis), and IHC subtype. Standardized differences were compared before and after adjusting for IPTW (Appendix Fig A1).^15^ All variables considered in IPTW showed adjusted standardized biases lower than 0.1, and their confounder effect was thus considered to be controlled by weight.^16^ Radiotherapy (yes/no) was added separately to the Cox model as the postsurgical covariable.

## RESULTS

### Clinical Characteristics of the MPBCS Cohort

**FIG A2:**
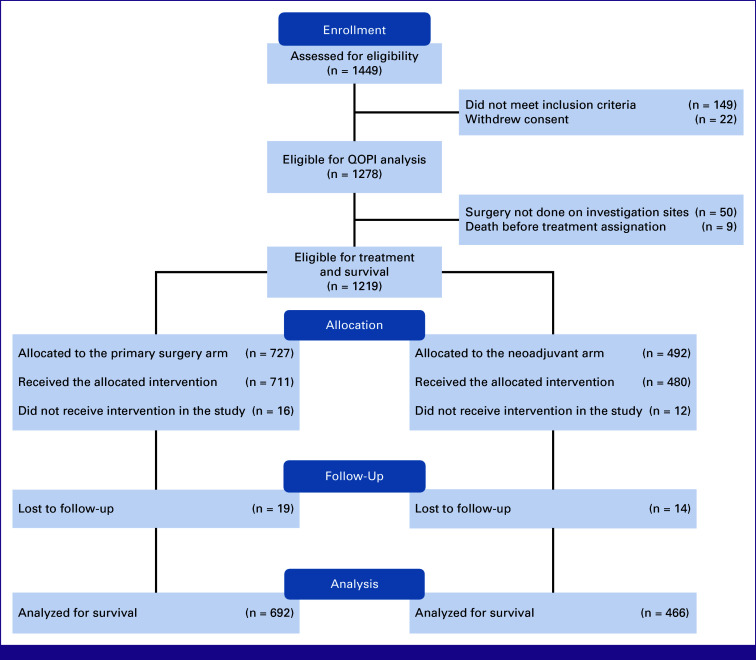
CONSORT diagram for MPBCS patients analyzed in this work. MPBCS, Molecular Profiling of Breast Cancer Study.

From a total of 1,449 recruited participants, 171 were excluded because of predefined exclusion criteria or consent withdrawal, leaving 1,278 eligible participants. For the survival analyses, 87 participants were further excluded because they did not receive treatment within the study, leaving a total of 1,191 participants. As a result, 711 and 480 participants were analyzed in primary surgery and neoadjuvant arms, respectively. Distribution of the participants is described in a CONSORT diagram (Appendix Fig A2).

Table 1 summarizes the baseline clinical and pathologic characteristics of MPBCS participants in primary surgery and neoadjuvant arms of the study, along with the type of surgery and NAC administration. NAC was considered complete if all the cycles were administered. Trastuzumab treatment was considered successful if at least one cycle was administered (Table 1). Overall, in the neoadjuvant arm, 97.1% of participants received at least one cycle of the drugs established per protocol and 37.9% received the complete scheme. In this arm, 62.2% of HER2+ participants received at least one dose of trastuzumab concomitant with NAC.

**TABLE 1:** Clinical, Pathologic, and Molecular Characteristics of Molecular Profiling of Breast Cancer Study Breast Cancer Participants by Arm of the Study (N = 1,191)

Arm	Primary Surgery (n = 711)	Neoadjuvant Chemotherapy (n = 480)	Total (N = 1,191)
Age, years, mean (SD)	12.1 (57)	10.8 (50)	
Age distribution, years, No. (%)			
<40	43 (6.0)	77 (16.0)	120
40-49	161 (22.6)	146 (30.4)	307
50-69	383 (53.9)	242 (50.4)	625
>69	124 (17.4)	15 (3.1)	139
Menopausal status, No. (%)			
Premenopausal	176 (27.8)	203 (47.5)	379
Perimenopausal	12 (1.9)	8 (1.9)	20
Postmenopausal	445 (70.3)	216 (50.6)	661
Missing	78	53	131 (11)
Histologic grade, No. (%)			
Low	106 (15.2)	47 (10.0)	153
Intermediate	297 (42.5)	245 (51.9)	542
High	296 (42.3)	180 (38.1)	476
Missing	12	8	20 (1.7)
cT stage, No. (%)			
T1	58 (8.4)	5 (1.0)	63
T2	576 (83.6)	127 (26.5)	703
T3	50 (7.3)	238 (49.7)	288
T4	5 (0.7)	109 (22.8)	114
Missing	22	1	23 (1.9)
cN stage, No. (%)			
N0	433 (62.6)	103 (21.5)	536
N1	245 (35.4)	267 (55.9)	512
N2	14 (2.0)	96 (20.1)	110
N3	0 (0.0)	12 (2.5)	12
Missing	19	2	21 (1.8)
IHC subtype, No. (%)			
HR+ HER2– Ki67-low	257 (38.5)	115 (24.7)	372
HR+ HER2– Ki67-high	212 (31.7)	128 (27.5)	340
HR+ HER2+	75 (11.2)	64 (13.8)	139
HR– HER2+	43 (6.4)	55 (11.8)	98
HR– HER2–	81 (12.1)	103 (22.2)	184
Missing	43	15	58 (4.9)
PAM50 subtype, No. (%)			
LumA	317 (49.4)	122 (31.1)	439
LumB	138 (21.5)	84 (21.4)	222
HER2E	69 (10.7)	69 (17.6)	138
Basal	84 (13.1)	86 (21.9)	170
Normal	34 (5.3)	31 (7.9)	65
Missing	69	88	157 (13)
Type of surgery, No. (%)			
Mastectomy	334 (50.8)	289 (72.8)	623
Quadrantectomy	323 (49.2)	108 (27.2)	431
Missing	54	83	137 (11.5)
Neoadjuvant therapy, No. (%)			480
Yes, complete		177 (37.9)	177
Yes, incomplete		289 (62.0)	289
Missing		14	14 (2.9)
Neoadjuvant trastuzumab, No. (%)			
HER2+ participants			119
Yes, any		74 (62.2)	74
No		45 (37.8)	45
Missing		0	0 (0)

NOTE. In categories with missing data, the percentages were calculated without considering missing data except for the total column in which the percentage of total missing for each variable is denoted.

Abbreviations: cN, clinical nodal status; cT, clinical T stage; HER2, human epidermal growth factor receptor 2; HR, hormone receptor; IHC, immunohistochemistry; SD, standard deviation.

Participants in the neoadjuvant arm tended to be younger or premenopausal (*P* < .001), with higher histologic grade (*P* = .002), larger tumor size, nodal involvement (*P* < .001), and higher frequency of nonluminal tumors (*P* < .001) than those in the primary surgery arm (Table 1).

**FIG A3:**
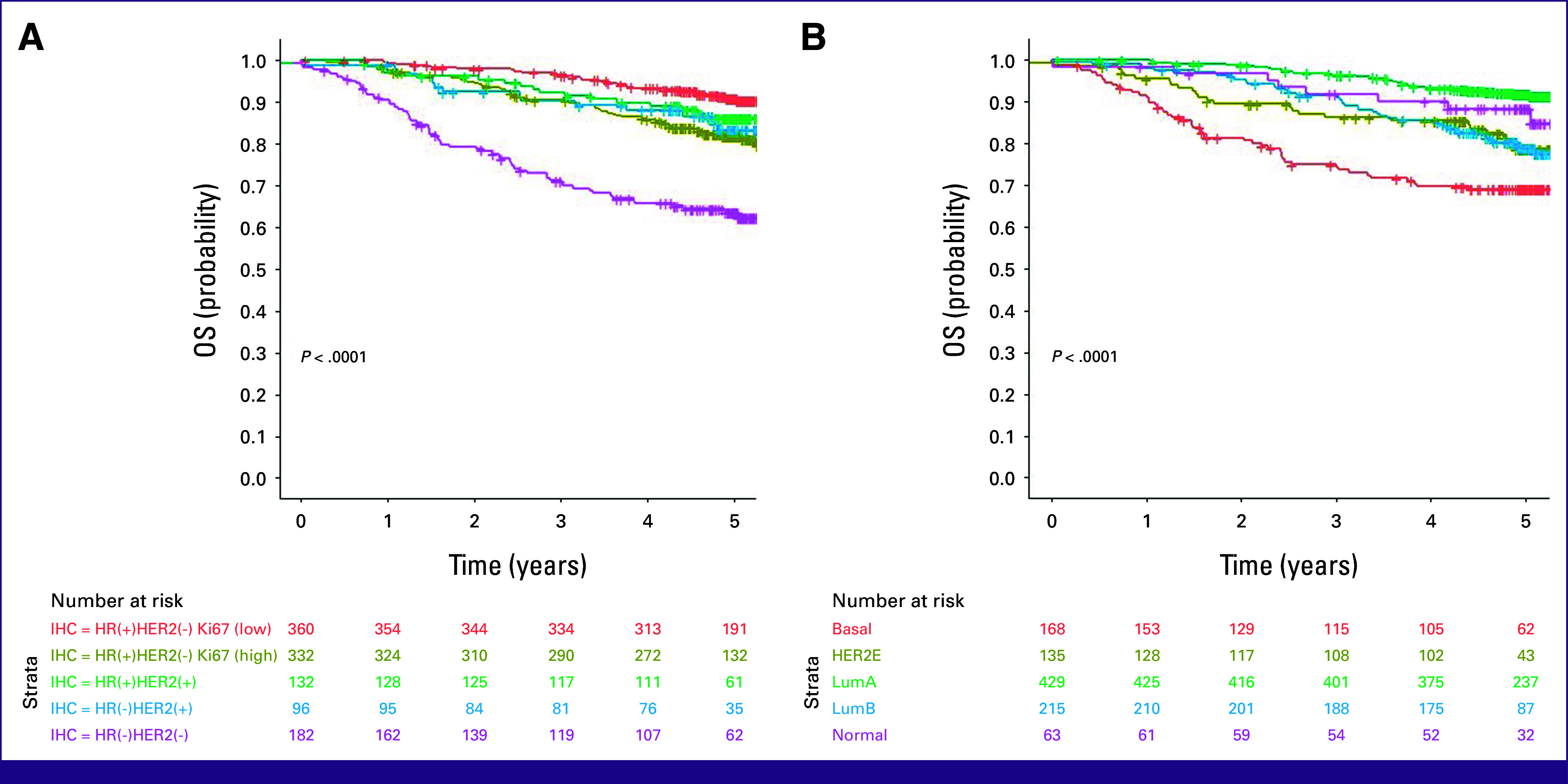
Kaplan-Meier analysis with the log-rank test of the OS of MPBCS participants according to their (A) immunohistochemical and (B) PAM50 subtypes. MPBCS, Molecular Profiling of Breast Cancer Study; OS, overall survival.

Cumulative survival probabilities at 5 years of 91.9% for PAM50-LumA, 79.0% for LumB, 78.8% for HER2E, and 69.0% for Basal-like participants were obtained for these participants (Appendix Fig A3). Among IHC-defined subtypes, HER2+ participants showed better survival than average PAM50-HER2E participants and hormone receptor (HR)+HER2+ participants showed slightly increased survival compared with HR-HER2+ participants (86.3% and 83.3%, respectively).

### RCB Cancer Burden as a Response to Neoadjuvant Treatment

**FIG 1:**
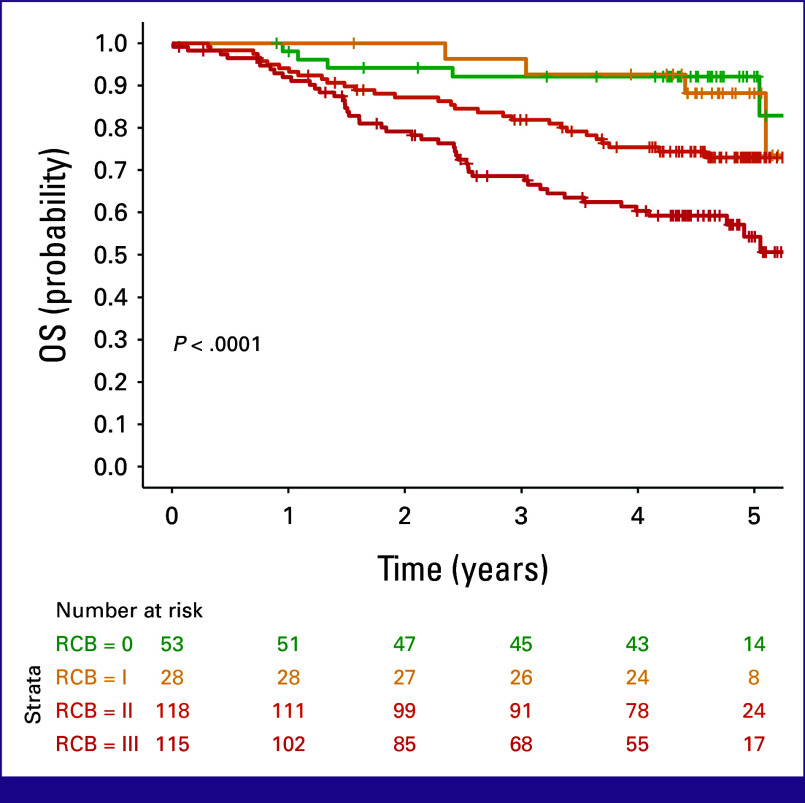
Kaplan-Meier analysis with the log-rank test of the overall survival of MPBCS patients assigned to the neoadjuvant arm, according to RCB. OS, overall survival; RCB, residual cancer burden.

OS of NAC participants was analyzed according to RCB, where RCB = 0 corresponds to pCR (Fig 1). Cumulative probabilities of 5-year survival were 92.1% for RCB and 0% and 88.2% for RCB I, decreasing to 63.0% for RCB II and 54.2% for RCB III (Fig 1). In Cox models, participants with RCB I were not statistically distinguishable from those with pCR in terms of survival, an observation that was preserved after adjustment for age, stage, PAM50 subtype, and type of surgery (Table 2). By contrast, RCB II and RCB III participants had significantly worse prognoses than RCB 0-I participants in both univariate and multivariate analyses (Table 2). After adjusting for confounding factors, participants with RCB II were 3.7 times more likely to die within 5 years than those with RCB 0, whereas those with RCB III were found to be 8.2 times more likely to die within the same time frame (Table 2).

**TABLE 2:** Cox Proportional Hazards Model of Overall Survival of Molecular Profiling of Breast Cancer Study Participants Assigned to the Neoadjuvant Arm, According to RCB

Variable	No.	Unadjusted Cox			Adjusted Cox		
		Hazard Ratio	95% CI	*P*	Hazard Ratio	95%	*P*
RCB							
RCB 0	53	reference			reference		
RCB I	28	1.40	0.4 to 5.3	.608	2.15	0.5 to 8.0	.272
RCB II	118	3.00	1.2 to 7.7	.023*	3.69	1.4 to 9.7	.008**
RCB III	115	5.40	2.1 to 13	<.001***	8.19	3.1 to 21	<.001***
Age	314	1.00	0.9 to 1.0	.844	0.99	0.9 to 1.0	.998
Menopausal status							
Premenopausal	154	reference			reference		
Perimenopausal	4	<0.001	0.0 to Inf	.995	<0.001	0.0 to Inf	.995
Postmenopausal	156	0.82	0.5 to 1.3	.373	0.83	0.4 to 1.6	.594
Grade							
Low	24	reference			reference		
Intermediate	163	2.60	0.8 to 8.4	.110	3.72	1.1 to 12	.038*
High	127	3.30	1.0 to 10	.048*	3.82	1.0 to 13	.041*
Country							
Argentina	95	reference			reference		
Brazil	50	1.12	0.6 to 2.1	.726	1.31	0.6 to 2.8	.489
Chile	54	1.16	0.6 to 2.1	.638	1.39	0.7 to 2.6	.313
Mexico	95	0.86	0.5 to 1.5	.605	0.69	0.4 to 1.3	.255
Uruguay	20	0.86	0.3 to 2.2	.750	0.79	0.3 to 2.2	.662
cT							
T1	4	reference			reference		
T2	83	0.61	0.1 to 4.6	.636	0.56	0.1 to 4.8	.592
T3	165	0.96	0.3 to 7.0	.971	0.85	0.1 to 7.0	.879
T4	62	1.10	0.1 to 8.2	.923	0.95	0.1 to 8.2	.961
cN							
N0	57	reference			reference		
N1	182	1.20	0.6 to 2.3	.527	0.76	0.4 to 1.5	.430
N2	69	2.30	1.2 to 4.6	.015*	1.23	0.6 to 2.6	.595
N3	6	1.80	0.4 to 8.2	.425	0.98	0.2 to 5.0	.983
IHC							
HR+ HER2– Ki67-low	71	reference			reference		
HR+ HER2– Ki67-high	92	1.50	0.8 to 3.0	.247	1.46	0.7 to 3.1	.321
HR+ HER2+	37	1.60	0.7 to 3.7	.313	2.13	0.9 to 5.2	.099
HR– HER2+	39	1.20	0.5 to 3.0	.746	1.81	0.6 to 5.0	.255
HR– HER2–	75	3.80	1.9 to 7.4	<.001***	5.12	2.5 to 10	<.001***

NOTE. Hazard ratios of univariate and multivariate models are shown with their corresponding 95% CIs and *P* values. Models considered RCB as the main predictor, and the rest of the variables as potential or known confounders or covariables. Analyses were performed only with the participants with no missing values in any of the variables involved. The asterisk denotes the level of statistical significance for the hazard ratio *P < .05, **P < .01, ***P < .001.

Abbreviations: cN, clinical nodal status; cT, clinical T stage; HER2, human epidermal growth factor receptor 2; HR, hormone receptor; IHC, immunohistochemistry; RCB, residual cancer burden.

**FIG 2:**
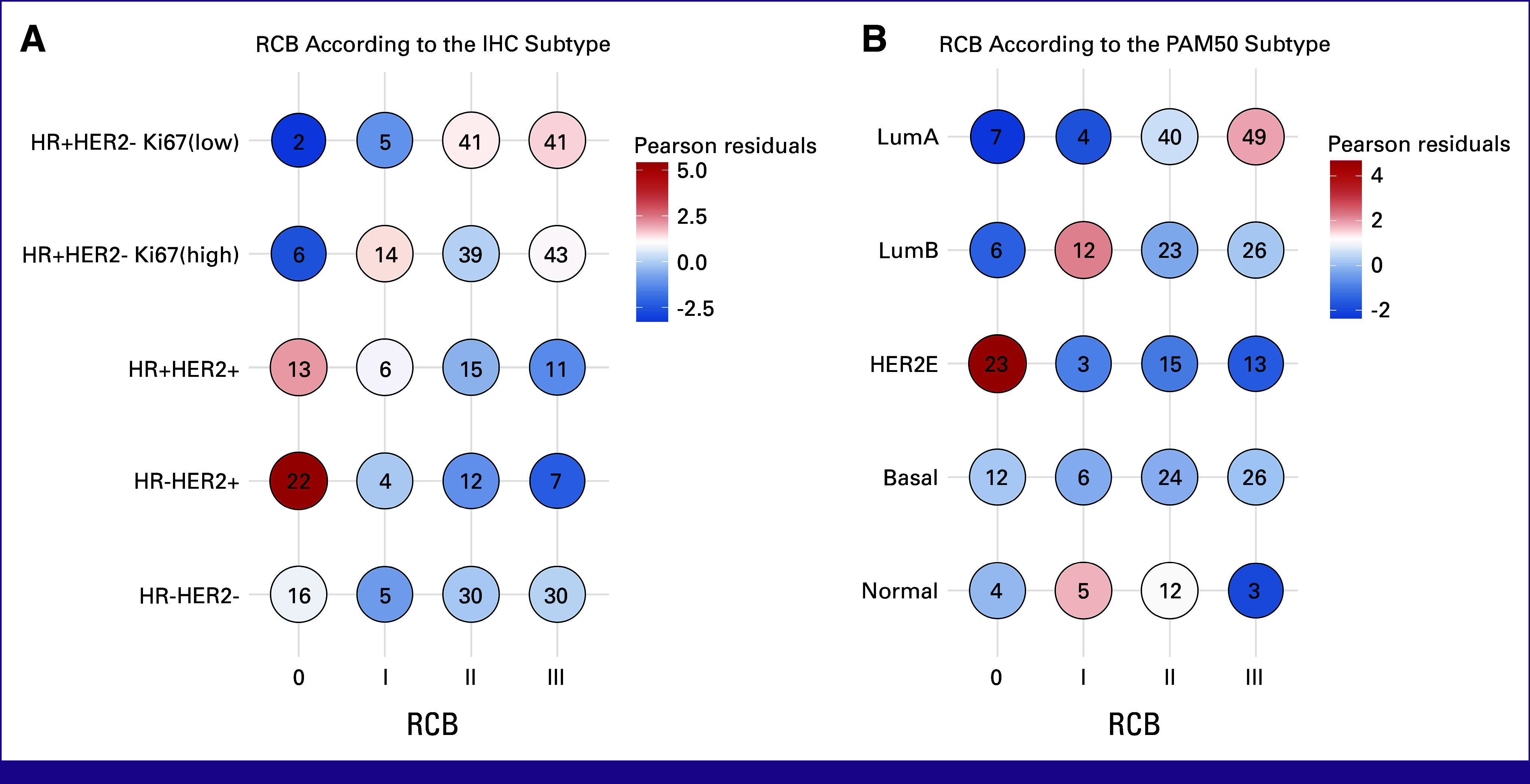
RCB according to the breast cancer subtype in the neoadjuvant arm of the MPBCS cohort. Distribution of RCB by immunohistochemical subtypes is shown in the left panel and by PAM50 subtypes in the right panel. Numbers within circles are the number of patients in each category. Positive Pearson residuals (red hues) specified an attraction (positive association), and negative residuals (blue hues) implied a repulsion (negative association) between the corresponding row and column variables. HER2, human epidermal growth factor receptor 2; HR, hormone receptor; IHC, immunohistochemistry; MPBCS, Molecular Profiling of Breast Cancer Study; RCB, residual cancer burden.

In addition, we found a significant association between RCB and IHC or PAM50 subtype (Fig 2). A good response (defined as RCB 0-I) was significantly associated with the HER2+ and HER2E subtypes. Basal tumors were also over-represented among good responders although this association was less significant than that observed in HER2+ cases. Consistently, poor responders (RCB II-III) were associated with HR+HER2– subtype (Fig 2, left).

When focusing on pCR, PAM50-HER2E tumors showed the highest level of pCR (42.6%), followed by Basal-like (18.0%), Luminal B (9.0%), and Luminal A (7.0%) subtypes (Fig 2, right). Similar tendency was observed when IHC subtypes were analyzed, where HR-HER2+ samples achieved the highest pCR rate (48.9%), followed by HR+HER2+ samples (28.9%) and triple-negative (TN) samples (20%; Fig 2). The HR+HER2– Ki67-low subtype had the lowest rate of pCR (4.2%), followed by HR+HER2-Ki67-high (5.9%; Fig 2, left).

We found no significant differences in pCR frequencies between countries (range, 8.9%-14.7%; Appendix Table A1).

### Type of Surgery and Survival

Among participants with the recorded surgery type, 40.9% underwent BCS (49.2% in the primary surgery arm and 27.2% in the neoadjuvant arm, Table 1). Most participants treated with NAC underwent total mastectomy (72.8%). Argentina and Chile showed a higher tendency to perform BCS in the neoadjuvant arm than the other countries, whereas Argentina, Chile, and Uruguay prioritized BCS in the primary surgery arm (Appendix Table A2).

**FIG 3:**
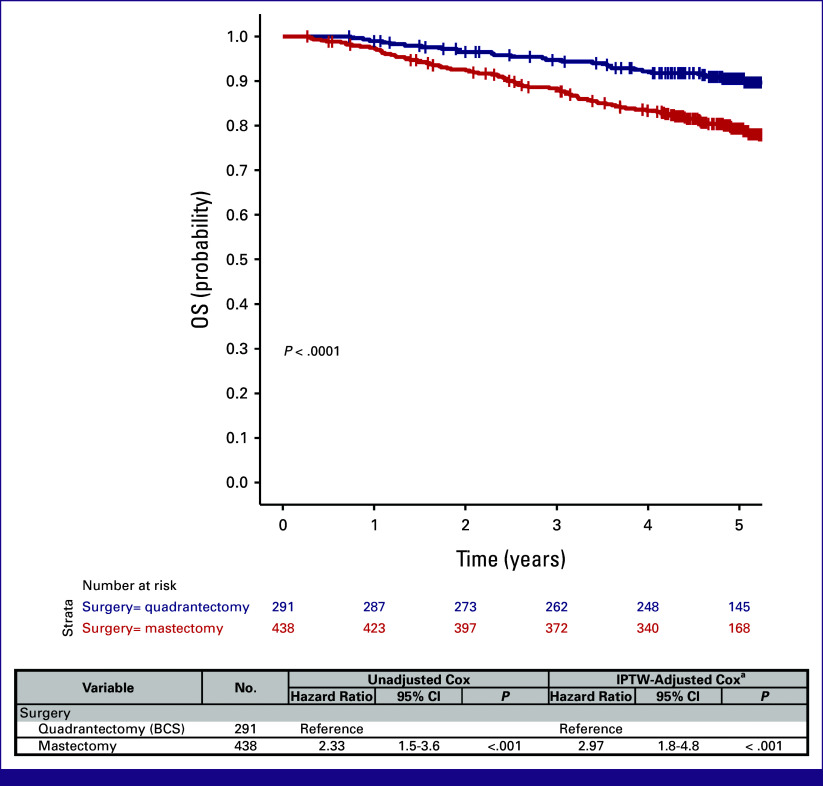
Overall survival of MPBCS patients according to the type of surgery (BCS or total mastectomy). Kaplan-Meier survival curves with log-rank analysis (above) and univariate and IPTW-corrected Cox proportional hazards regression model hazard ratios with their correspondent 95% CIs and *P* values (below). ^a^The model considered (1) the type of surgery as the main predictor of survival, (2) variables that affect the type of surgery decision as weights from IPTW analysis, and (3) radiotherapy (yes/no) as a covariable. Analyses were performed only with the patients with no missing values in any of the variables involved. BCS, breast-conserving surgery; IPTW, inverse probability of treatment weighting; MPBCS, Molecular Profiling of Breast Cancer Study; OS, overall survival.

To evaluate survival outcomes on the basis of the type of surgery, we focused on the entire cohort, encompassing both up-front and postprimary systemic therapy surgery. Using a causal approach (IPTW) to control for confounders, we found that survival was significantly worse for those undergoing total mastectomy compared with BCS (unadjusted hazard ratio = 2.3, IPTW-adjusted hazard ratio = 3.0, Fig 3), indicating that women who underwent total mastectomy had a three-fold higher risk of death compared with those who underwent BCS, even after accounting for variables influencing surgical decisions and outcome (ie, age, menopausal status, grade, country, neoadjuvant treatment, cT, cN, pT, IHC subtype, and radiotherapy).

### Analysis of QOPI Breast Module Standards in the MPBCS Cohort

Complete staging and HER2/neu testing (BR52a and BR54) were achieved in more than 90% of participants, indicating remarkable overall compliance (ie, higher than 75%; Table 3). By contrast, treatment administration (BR53, BR55/57, and BR58/59) achieved lower-quality standards, with the poorest results for trastuzumab access and timely hormone therapy.

**TABLE 3:** QOPI Standards for Breast Cancer Care in the Molecular Profiling of Breast Cancer Study Cohort (n = 1,278)

Country	Argentina	Brazil	Chile	Mexico	Uruguay	Total
QOPI standard, No./Total (%)						
BR52a: complete staging for patients with invasive breast cancer (cancer stage, HER2, and ER/PR status)	251/254 (98.8)	267/284 (94.0)	159/175 (90.8)	391/455 (85.9)	100/110 (90.9)	1,168/1,278 (91.4)
BR53: combination chemotherapy received within 4 months of diagnosis by women younger than 70 years with AJCC stage IA (T1c) and IB-III ER-/PR-negative breast cancer	26/36 (72.2)	42/51 (82.3)	22/25 (88.0)	88/98 (89.8)	11/11 (100)	189/221 (85.5)
BR54: test for HER-2 overexpression or gene amplification	252/254 (99.2)	270/284 (95.1)	161/175 (92.0)	410/455 (90.1)	103/110 (93.6)	1,200/1,278 (93.9)
BR57 (also BR55): Appropriate treatment for patients with stage I (T1c)-III HER2-positive breast cancer	39/44 (88.6)	55/62 (88.7)	9/27 (33.3)	55/99 (55.5)	9/14 (64.3)	167/246 (67.9)
BR58 (also BR59): tamoxifen or AI received within 1 year of diagnosis by patients with AJCC stage IA (T1c) and IB-III ER-/PR-positive breast cancer	64/183 (34.9)	77/215 (35.8)	31/128 (24.2)	103/280 (36.8)	40/81 (49.4)	315/887 (35.5)^a^
BR58 by study arm—primary surgery arm, No./Total (%)	38/88 (43.2)	38/76 (50.0)	25/64 (39.1)	67/126 (53.2)	34/49 (69.4)	202/567 (35.6)^a^
BR58 by study arm—neoadjuvant arm, No./Total (%)	26/68 (38.2)	39/57 (68.4)	6/35 (17.1)	36/75 (29.2)	6/15 (40.0)	113/320 (35.3)^a^

NOTE. This table only includes the QOPI's breast cancer–specific standards that could be evaluated in the MPBC study. The BR58 standard was also calculated by arm to show the by-country effect of neoadjuvancy in the delay of tamoxifen or AI administration.

Abbreviations: AJCC, American Joint Committee on Cancer; ER, estrogen receptor; HER2, human epidermal growth factor receptor 2; PR, progesterone receptor; QOPI, Quality Oncology Practice Initiative.

^a^ The denominator includes patients with no registry of treatment, with antihormonal therapies other than tamoxifen or AI, and/or with no date of treatment initiation. The total of patients who have a registered tamoxifen or AI adjuvant treatment at any moment is 653 of 887 for the whole cohort, 403 of 567 for the primary surgery arm and 250 of 320 for the neoadjuvant arm.

At least one dose of trastuzumab was administered to 67.9% of HER2+ participants, but remarkable variations among countries were observed, ranging from 33.3% in Chile to more than 88% in Brazil and Argentina.

Regarding the interval between diagnosis and therapy initiation, high compliance with this indicator was observed for TN participants (BR53), with 85.5% of participants receiving treatment within 4 months of diagnosis. Again, differences between countries were observed, with Argentina exhibiting the lowest percentage (72.2%) versus 100% of participants in Uruguay (Table 3). In addition, interval between diagnosis and initiation of treatment with adjuvant tamoxifen or aromatase inhibitors (AI) in HR+ participants (BR58/BR59) was below standard, with only 35.5% of the participants receiving tamoxifen or AI within 1 year of diagnosis. Interestingly, Chile exhibited the lowest compliance, with 24.2% of participants receiving treatment at due time, compared with 49.4% in Uruguay. When comparing participants who received tamoxifen or AI at the expected time with the total number of participants who received tamoxifen or AI at any time, compliance increased to 48.2%. In all countries except Brazil, delays in treatment initiation were worse in the neoadjuvant than in the primary surgery arm (Table 3).

## DISCUSSION

To our knowledge, MPBCS is the first multicountry and comprehensive research initiative in LA designed to characterize distribution of molecular profiles, epidemiologic factors, clinicopathologic characteristics, and participant outcomes in LABC.^8,13^ This study implemented harmonized standard activities addressing systemic treatments, pathology (including FISH analysis of HER2 on specimens), response assessment, state-of-the-art biobanking (including first-time biobanking for some public hospitals), anti-HER2 therapies, and molecular subtype classification to guide treatments.

As expected for a LABC cohort, more aggressive subtypes (ie, PAM50-LumB, HER2E, and Basal-like) were observed; this pattern aligns with various hospital-based studies on Hispanic/Latina women.^17-19^ The 5-year OS in this cohort is consistent with other studies in LA countries^20^ and represents an improvement over historical data.^1^

Different meta-analyses have confirmed that participants with pCR have better OS and disease-free survival than those with residual tumors.^21-23^ In our cohort, prognostic value of RCB scores (including pCR) was confirmed. OS-adjusted hazard ratio between participants who achieved pCR and those who did not was equivalent to adjusted hazard ratios described by Spring et al^24^ and a study in Colombia.^25^ Moreover, cumulative probabilities of survival according to each RCB score observed in our study were consistent with those reported by Yau et al.^26^ We did not detect significant differences between pCR and RCB I, likely because of our smaller RCB I group size; however, a good response still offers a survival advantage over a limited or no response.

According to the meta-analyses mentioned above, a wide variation was observed in percentage of participants of each molecular subtype that achieved pCR.^21-23^ However, in all studies, subtype with the highest percentage of pCR was HER2+, particularly HR-HER+. In our cohort, although only two thirds of HER2+ participants received trastuzumab, they exhibited the highest pCR rates. Conversely, HR+HER2– participants had the lowest pCR rates, consistent with literature findings. Interestingly, despite similar NAC treatments, our cohort's pCR rate for TN participants (20%) was lower than that previously reported (32.6%-43.0%),^21-23^ suggesting that factors beyond the chemotherapy regimen influenced pCR rates.

Regarding the type of surgery, our findings suggest that BCS might be associated with better OS than mastectomy in participants with LABC. This observation remains consistent even after adjusting for pretreatment confounders (age, menopausal status, stage, grade, tumor size, involved nodes, subtype, previous neoadjuvant treatment, and country) and post-treatment covariables, such as radiotherapy, indicating that the type of surgery might have a prognostic value independent of those variables. We cannot completely discard factors other than those controlled, such as participant's decision (which was not recorded in this study), which might play a role in this effect. Nevertheless, this observation aligns with data from other observational studies from Netherlands Cancer Registry,^27^ SEER database,^28^ and Swedish national database^29^ and might reflect improvement in BCS techniques. Accumulating evidence underscores that BCS should be considered whenever feasible and emphasizes the need to critically evaluate factors influencing surgical decisions, especially given the reported lack of access to specialized surgical services in the LA region.^30-32^

Our analysis of specific key indicators recommended by ASCO showed that access to standard-of-care procedures was constrained in a real-life setting, notably in the use of trastuzumab. While trastuzumab has been covered in Uruguay since 2006^33^ and Argentina since 2006,^34^ its inclusion in Chile's health care system only occurred in 2015.^35^ This later inclusion likely contributed to the lower rate of trastuzumab use observed. Furthermore, neoadjuvant setting presented additional challenges, as in some countries, coverage was initially limited to adjuvant treatments.^36^ These factors collectively might explain why only 37.9% of patients the in NAC group received the complete chemotherapy scheme and the low usage of trastuzumab. In addition, all members of LACRN encountered barriers to compliance with other QOPIs, particularly for HR+ participants who did not receive timely adjuvant hormone therapy or did not receive it at all. NAC also prolonged the time to surgery, leading to more frequent noncompliance with timely access to adjuvant hormone therapy. For this standard, it is also relevant that some participants received other antihormone treatments (ie, luteinizing hormone-releasing hormone [LH-RH] agonist [n = 88] and oophorectomy [n = 2]), although these were not considered for BR58/59 standard.

This study has limitations. First, our capacity to test the significance of differences in RCB, particularly pCR, by subtype or across countries, was constrained. The limited number of participants in each category did not allow for the statistical power necessary to detect significant differences. This limitation points to the need for larger multiregional studies to robustly evaluate these aspects. Furthermore, our analysis of surgical outcomes, notably the comparison between BCS and mastectomy, was limited by the lack of more comprehensive data. Critical factors such as expertise of the surgeons, specifics of surgical margins, and reasons guiding the choice of surgical technique were not included in our data set. Another limitation was the variability in access to essential treatments across participating Latin American countries, which was not recorded for this study. This inconsistency, attributed to diverse health care policies, likely affected adherence to treatment protocols and overall study outcomes.

To fully understand and account for noncompliance with treatment-related standards and to address the limitation by variables not collected in the study, we decided to amend the protocol to obtain additional follow-up data. This amendment aims to specifically collect data on patterns of care and access to diagnostic and treatment procedures.

In summary, this study has provided essential insights into the clinical characteristics and treatment outcomes of LABC across LA. Despite encountering limitations, including variability in access to key treatments, this study represents a significant step forward in understanding the real-world implementation of oncologic procedures in diverse health care environments. The findings underscore the need for harmonized and improved access to essential treatments throughout the region.

## APPENDIX

**TABLE A1:** Distribution of RCB Among Countries of Participants in the Neoadjuvant Arm

RCB	Country				
	Argentina, No. (%)	Brazil, No. (%)	Chile, No. (%)	Mexico, No. (%)	Uruguay, No. (%)
0	15 (12.9)	10 (8.9)	8 (13.1)	21 (13.4)	5 (14.7)
I	9 (7.8)	7 (6.2)	8 (13.1)	10 (6.4)	0 (0.0)
II	45 (38.8)	26 (23.2)	21 (34.3)	38 (24.2)	9 (26.5)
III	47 (40.5)	17 (15.2)	19 (31.1)	44 (28.0)	8 (23.5)
Missing	0 (0.0)	52 (46.4)	5 (8.2)	44 (28.0)	12 (35.3)

Abbreviation: RCB, residual cancer burden.

**TABLE A2:** Type of Surgery Distribution by Arm and Country (n = 1,054)

Arm	Primary Surgery				
Country	Argentina, No. (%)	Brazil, No. (%)	Chile, No. (%)	Mexico, No. (%)	Uruguay, No. (%)
Mastectomy	44 (34.9)	83 (56.5)	47 (43.1)	145 (63.3)	15 (32.6)
BCS	82 (65.1)	64 (43.5)	62 (56.9)	84 (36.7)	31 (67.4)

Arm	Neoadjuvant Chemotherapy				
Country	Argentina, No. (%)	Brazil, No. (%)	Chile, No. (%)	Mexico, No. (%)	Uruguay, No. (%)
Mastectomy	52 (53.6)	84 (87.5)	30 (50)	109 (85.2)	14 (87.5)
BCS	45 (46.4)	12 (12.5)	30 (50)	19 (14.8)	2 (12.5)

Abbreviation: BCS, breast-conserving surgery.
